# Translational Control of Canonical and Non-Canonical Translation Initiation Factors at the Sea Urchin Egg to Embryo Transition

**DOI:** 10.3390/ijms20030626

**Published:** 2019-02-01

**Authors:** Héloïse Chassé, Sandrine Boulben, Patrick Cormier, Julia Morales

**Affiliations:** Centre National de la Recherche Scientifique (CNRS), Sorbonne Université, Integrative Biology of Marine Models (LBI2M), Station Biologique de Roscoff (SBR), 29680 Roscoff, France; hchasse@sb-roscoff.fr (H.C.); boulben@sb-roscoff.fr (S.B.); cormier@sb-roscoff.fr (P.C.)

**Keywords:** sea urchin fertilization, translational control, initiation complex, eIF4E, mTOR pathway

## Abstract

Sea urchin early development is a powerful model to study translational regulation under physiological conditions. Fertilization triggers an activation of the translation machinery responsible for the increase of protein synthesis necessary for the completion of the first embryonic cell cycles. The cap-binding protein eIF4E, the helicase eIF4A and the large scaffolding protein eIF4G are assembled upon fertilization to form an initiation complex on mRNAs involved in cap-dependent translation initiation. The presence of these proteins in unfertilized and fertilized eggs has already been demonstrated, however data concerning the translational status of translation factors are still scarce. Using polysome fractionation, we analyzed the impact of fertilization on the recruitment of mRNAs encoding initiation factors. Strikingly, whereas the mRNAs coding eIF4E, eIF4A, and eIF4G were not recruited into polysomes at 1 h post-fertilization, mRNAs for eIF4B and for non-canonical initiation factors such as DAP5, eIF4E2, eIF4E3, or hnRNP Q, are recruited and are differentially sensitive to the activation state of the mechanistic target of rapamycin (mTOR) pathway. We discuss our results suggesting alternative translation initiation in the context of the early development of sea urchins.

## 1. Introduction

Regulation of protein synthesis is critical for cell growth, development and survival and is now recognized as a key control of gene expression [1,2]. Translation is predominantly regulated during the initiation step. The majority of eukaryotic mRNAs are translated using a cap-dependent mechanism: eukaryotic initiation factors (eIFs) assemble on the 5’ cap structure of the mRNA to recruit the 43S preinitiation complex which scans mRNA in a 3’ direction to locate the AUG start codon and facilitate 60S subunit joining, forming a translationally active 80S ribosome [3]. The initiation complex includes the cap-binding protein (eIF4E), the scaffold protein (eIF4G), and an RNA helicase (eIF4A) [4]. The translation initiation factor eIF4B potentiates eIF4A RNA helicase activity in vitro [5]. The poly(A)-binding protein (PABP) bridges the 5’ and 3’ ends of the mRNA by binding to eIF4G and the poly(A) tail of the mRNA, conferring a closed-loop conformation to the mRNA, that increases translation efficiency [6]. The small protein 4E-BP competes with eIF4G for the binding domain (YX_4_LΦ) of eIF4E [7] and consequently is able to inhibit translation initiation. The phosphorylation status of 4E-BP is regulated by the mTOR (mechanistic target of rapamycin) pathway and controls its association with eIF4E [8]. Cell proliferation is tightly regulated by translational control and has been shown to involve the eIF4E-dependent translation of proto-oncogenes [9]. The mRNAs of proto-oncogenes have structured untranslated regions that regulate cap-dependent translation initiation through helicase-mediated unwinding of RNA structures [10,11]. Furthermore, overexpression of translation factors leads to proliferation and cellular transformation. The mTOR and MAPK/ERK (mitogen-activated protein kinase) pathways target several translation factors to regulate protein synthesis [12]. As a major effector of cell growth and proliferation, mTOR is a favorite target for therapeutic inhibition. However, mTOR pathway inhibition can lead to a hyperactivation of the MAPK pathway in mammalian cells [13], both pathways being connected by compensatory feedback loops [14]. Deciphering mRNA targets of the different pathways will allow a better comprehension of the molecular circuitry impacting translational control.

The eIF4E family is composed of three classes represented by eIF4E1, eIF4E2, and eIF4E3, which share ~30% identity [15,16]; eIF4E1 has been found in all eukaryotes; eIF4E2 in all metazoans; and eIF4E3 in deuterostomes [15,16], including sea urchins [17]. eIF4E1 is the canonical cap-binding protein implicated in translation initiation, whereas eIF4E2 and eIF4E3 are involved in mRNA-specific translation regulation [18,19,20,21]. In some specific physiological or physio-pathological cases, alternative cap- and/or eIF4E1-independent translation can occur, in which non-canonical translation factors take control [22]. DAP5 is a protein homologous to the C-terminal end of eIF4G that is unable to interact with eIF4E or PABP but still retains the capacity to bind eIF3 and eIF4A. DAP5 can mediate translation of its own mRNA by the direct recruitment of ribosomes at the vicinity of the ORF start codon on IRES (internal ribosome entry site) [23] as well as for the translation of a subset of mRNAs [24,25,26]. Recently, DAP5 was shown to also affect translation of mRNAs that do not contain IRES [26]. New members of the poly(A)-binding family have been characterized recently, among them hnRNP Q (also termed Syncrip) [27]. This protein, a competitive inhibitor of PABP binding, acts as a translation inhibitor [28] but also promotes IRES-dependent translation of specific mRNAs [29,30].

Sea urchin early development is a model in which to study translation and cell cycle regulation. Indeed, the first cell cycles of the sea urchin embryo depend on the translational up-regulation that occurs at fertilization [31,32]. The sea urchin genome contains full repertoire of known translation factors, each one encoded by a unique gene [17,33]. In unfertilized eggs, translation activity is low. Fertilization triggers an increase in protein synthesis dependent upon the activation of the translation machinery and the polysomal recruitment of the stored maternal mRNAs, independently of transcription [34,35,36]. The increase in protein synthesis triggered by the fertilization of sea urchin eggs is partly explained by an increase in the activity of the cap-binding complex [37,38,39]. The cap-dependent translation inhibitor 4E-BP plays an important role in eIF4E sequestration in unfertilized eggs. Following fertilization, 4E-BP is rapidly phosphorylated and degraded in an mTOR-dependent manner, leading to the release of eIF4E [40,41,42]. eIF4E is available to associate with eIF4G [43]. Therefore, eIF4E is now recognized as a crucial actor for the onset of the first mitotic division following fertilization, suggesting that cap-dependent translation is highly regulated during the egg-to-embryo transition in sea urchins [44]. Interestingly, when performed in the presence of the mTOR inhibitor PP242, a differential recruitment of maternal mRNAs to polysome was observed after fertilization. While several mRNAs showed sensitivity to the inhibitor, indicative of recruitment via the canonical cap and eIF4E recognition, other mRNAs are still recruited to the polysomes in the presence of the inhibitor, suggesting that an alternative mode of translation is also functional at fertilization [45]. Furthermore, PP242 has been identified as an ERK activator in multiple myeloma cells [46], raising the question of whether a similar relay could operate physiologically in the developing sea urchin embryo.

Besides the post-translational regulation and protein interactions described above, an additional potential layer of regulation of the protein machinery is the translational control of mRNAs encoding the translation factors. The study of the translational status of specific mRNAs by analysis of polysome localization allows the direct assessment of mRNAs recruited after fertilization [47]. In this report, we focused on the polysomal recruitment of mRNAs for canonical and non-canonical factors involved in mRNA recruitment at the initiation step of translation.

## 2. Results

### 2.1. mRNA Coding Translation Initiation Factors Involved in mRNA Recognition Are Present as Maternal mRNAs in Sea Urchin Eggs

An antibody directed to the human full-length protein eIF4E1 cross-reacts with two bands at 24–25 kDa, the lower band being the most prominently associated to an m^7^-GTP column, revealing the existence of cap-binding protein in sea urchin eggs [40]. Whether the upper band revealed by western blot corresponds to eIF4E1, eIF4E2, or eIF4E3 is currently unknown. Information on mRNA abundance of the translation factors was derived from transcriptome analysis of maternal mRNAs using FPKM values as an indicator of mRNA abundance [45]. Based on FPKM values, mRNAs encoding eIF4E1 were present at very low levels (<1 FPKM) in the maternal transcriptome. In contrast, the mRNAs for the helicase eIF4A, the scaffolding protein eIF4G, the initiation factor eIF4B, and the poly(A)-binding protein (PABP) gave significantly higher FPKM values from 6.5 to >94. FPKM values and the primers used for PCR amplification are presented in Table 1.

### 2.2. Initiation Factors eIF4A, eIF4G, or PABP Are not Recruited into Polysomes after Fertilization

**Figure 1:**
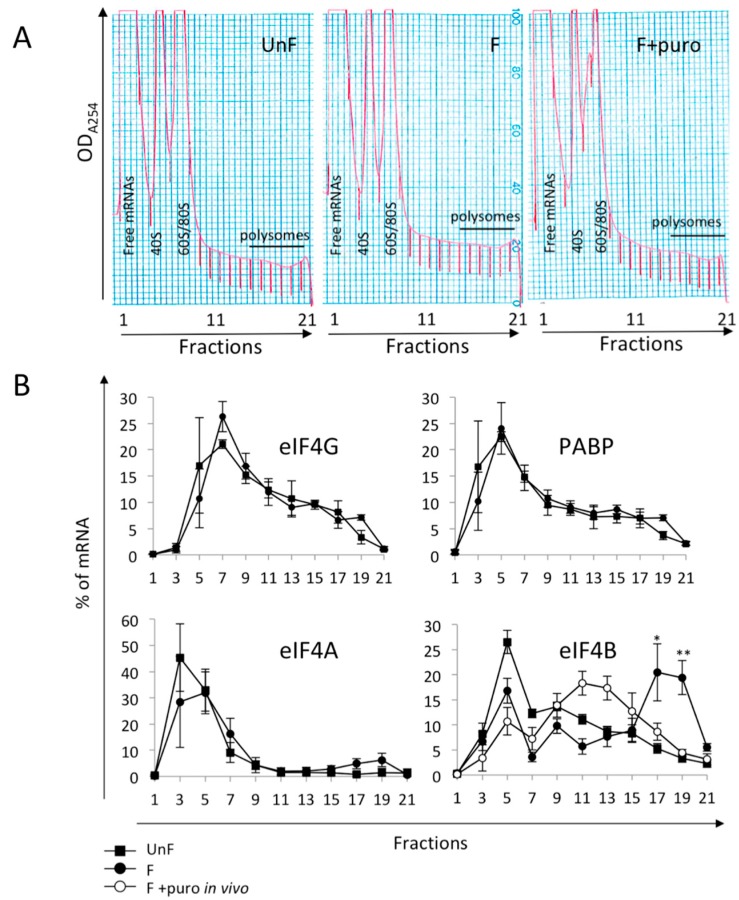
(**A**) Optical density profiles (OD_A254_) of polysome fractionation from unfertilized eggs (UnF), embryos at 1 h post-fertilization (F) and in presence of puromycin (F+puro). Fractions 1 and 21 correspond to top and bottom, respectively, of the 15–40% sucrose gradient; (**B**) Distribution in polysomes of mRNAs encoding initiation factors eIF4G, eIF4A, eIF4B, and poly(A)-binding protein (PABP) before fertilization (UnF, square), at 1 h post-fertilization (F, black dot), and at 1 h post-fertilization in presence of puromycin (F+puro in vivo, white dot). mRNAs were detected by RT-PCR of RNA purified from each fraction of the gradient. Amplified products were separated on agarose gel and quantified as described in the Materials and Methods section. Distribution is shown as a percentage of total mRNA (*n* = 5; UnF vs. F: * *p*-value < 0.05, ** *p*-value < 0.01).

We first asked whether mRNAs for the initiation factors involved in the cap recognition were recruited into polysomes after fertilization. We analyzed the distribution of these mRNAs on polysome gradients in unfertilized eggs and in 1-hour post-fertilization embryos, after 4E-BP degradation and eIF2α dephosphorylation but before entry into mitosis, which could impact translational activity [44]. Polysome profiles, monitored by the A_254_ scan of the fractionated gradient (Figure 1A), showed three peaks in the light fractions, corresponding to free mRNAs, 40S, and 60S/monosomes. The polysomes are localized in the heavy fractions (15–21), as described previously [45,47].

In agreement with the low level of mRNAs for eIF4E1 present in the transcriptome, eIF4E1 mRNAs were not detectable on polysome fractions by RT-PCR of RNA from unfertilized eggs or from embryos at 1 h post-fertilization. As shown in Figure 1B, mRNAs for eIF4A, eIF4G, and PABP were mostly present in the light fractions (1–7) of the gradient, and their distribution is not modified after fertilization, suggesting no increase in their recruitment. In contrast, eIF4B mRNAs are present in the light fractions (1–7) of the gradient before fertilization and are associated with the heavy fractions (17–21) after fertilization. Upon puromycin treatment, which disrupts only actively translating polysomes but not co-migrating mRNPs, the eIF4B mRNAs shift to the middle of the gradient (fractions 9–13), suggesting that eIF4B is translationally regulated at fertilization. These results are in agreement with the translatome data analysis at the egg-to-embryo transition [45].

### 2.3. Non-Canonical Initiation Complex mRNAs Are Present in Unfertilized Eggs and Translated at Fertilization

Each canonical initiation factor involved in the mRNA activation possesses a non-canonical counterpart (described in Table 2) that can act as a specific translation factor or inhibitor for selective translation. The mRNAs for eIF4E2 and eIF4E3 were present on the transcriptome and detectable by RT-PCR, in contrast to eIF4E1 mRNAs. The mRNAs coding DAP5, a truncated homolog of eIF4G, and the mRNAs coding hnRNP Q, a protein able to interact with poly(A), were detected also in the maternal transcriptome in amounts exceeding the canonical counterpart (Table 1).

**Figure 2:**
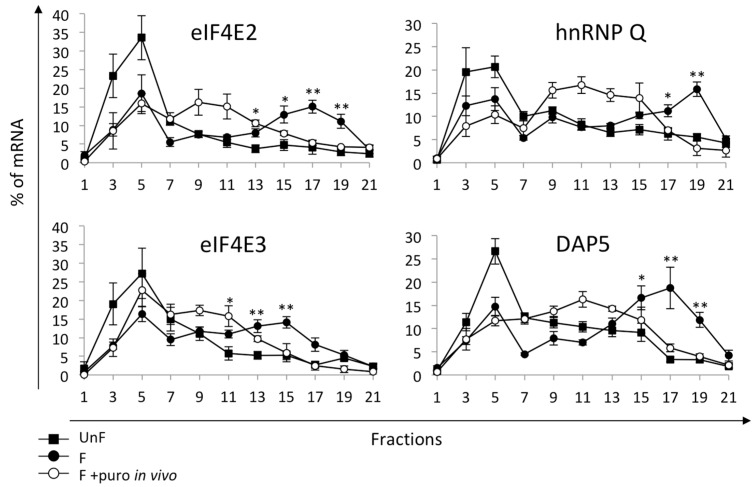
Distribution of mRNAs for eIF4E2, eIF4E3, hnRNP Q and DAP5 in polysomes, monitored as in Figure 1 (*n* = 5; UnF vs. F: * *p*-value < 0.05, ** *p*-value < 0.01).

In addition, we asked whether these mRNAs were translationally regulated (Figure 2). Before fertilization, eIF4E2 and eIF4E3 mRNAs sedimented in the light fractions (1–7) of the gradient and after fertilization shifted towards the heavy fractions (15–21). For eIF4E2, the mRNA was present in fractions 15 to 21 and was displaced to the middle of the gradient when embryos were treated with puromycin. For eIF4E3, the mRNAs were associated to smaller polysomes, peaking at fraction 15, and were also displaced by puromycin treatment. These data demonstrate that the two members of the eIF4E family, namely eIF4E2 and eIF4E3, were actively recruited after fertilization. The mRNAs for eIF4E2 and eIF4E3 were found in lighter fractions than mRNA for eIF4B, suggesting a lower recruitment rate. This localization explains why these mRNAs were not detected in the earlier transcriptome analysis, which focused on recruitment in polysome fractions 18 to 21 at fertilization [45]. In contrast, DAP5 is strongly recruited into heavy polysomes after fertilization and displaced by puromycin treatment (Figure 2), indicative of active recruitment after fertilization in agreement with the translatome data [45]. Distribution in polysome of the hnRNP Q mRNA showed that it is also recruited into fractions 17–21 of the gradient after fertilization, and it is displaced by puromycin treatment, indicating that hnRNP Q is actively recruited after fertilization.

Altogether, among the maternally stored mRNAs in sea urchin eggs, we demonstrated that eIF4B, eIF4E2, eIF4E3, DAP5, and hnRNP-Q mRNAs are recruited into polysomes and translated after fertilization, whereas mRNAs for eIF4A, eIF4G, and PABP were not.

### 2.4. mTOR Pathway Differentially Impacts the Polysomal Recruitment of Initiation Factor mRNAs

**Figure 3:**
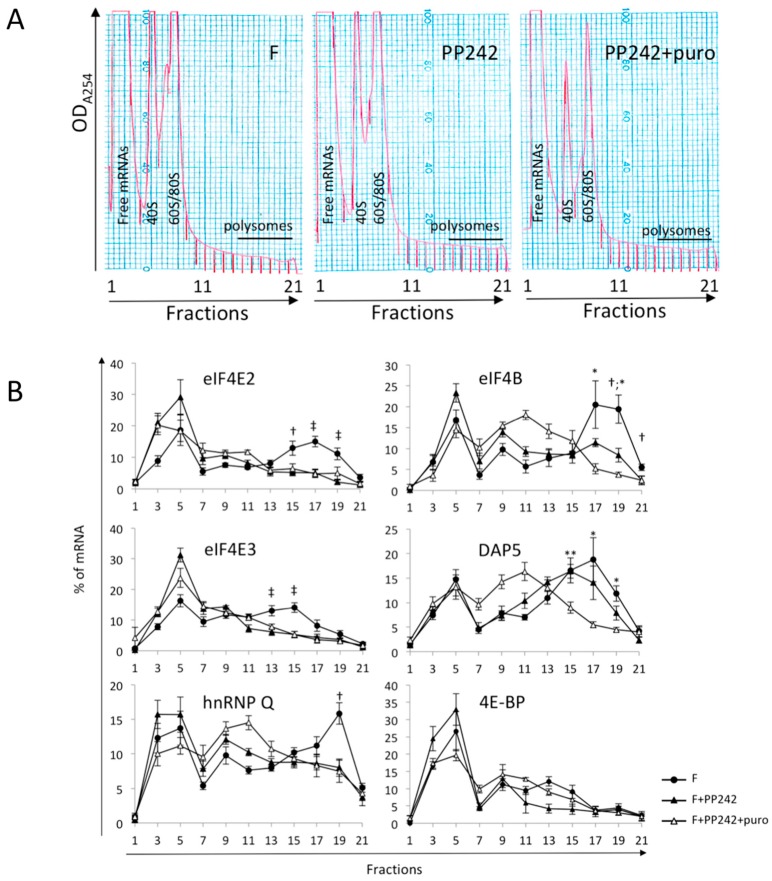
(**A**) Polysome profile of fertilized embryos (F) with or without PP242 inhibitor and in presence of puromycin (F+puro). (**B**) mTOR impact on polysomal recruitment of mRNAs encoding translation initiation factors after fertilization. Localization of the targeted mRNAs along the gradient is monitored as in Figure 1. Samples were treated or not with PP242 and puromycin (*n* = 5; F vs. F+PP242: † *p*-value < 0.05, ‡ *p*-value < 0.01; F+PP242 vs. F+PP242+puromycin: * *p*-value < 0.05, ** *p*-value < 0.01).

In order to study the possible involvement of the mTOR pathway on polysomal recruitment, we investigated the effect of the mTOR active site inhibitor, PP242, on mRNA recruitment in embryos at 1 h post-fertilization. Upon PP242 treatment, the mRNAs for eIF4E2, eIF4E3, and hnRNP Q completely shifted from heavy fractions (15–21) of the polysome gradient to the light fractions (Figure 3). The polysomal distribution was similar in PP242-treated embryos and those treated with both PP242 and puromycin, indicating that all mRNAs were released from polysomes upon mTOR inhibition. These results suggested that entry into polysomes at fertilization was completely dependent on the mTOR pathway for eIF4E2, eIF4E3, and hnRNP Q mRNAs. As expected [45], DAP5 mRNA recruitment was similar in control and in PP242-treated embryos. Recruitment of eIF4B mRNA was only partially inhibited by PP242 treatment (Figure 3). The puromycin treatment performed on PP242-treated embryos shifted the remaining DAP5 and eIF4B mRNAs completely towards the middle of the gradient, suggesting that the mRNAs remaining in polysome when mTOR is inhibited were still actively translated.

We next asked whether mTOR inhibition would impact 4E-BP translation. The 4E-BP protein remains present in PP242-treated embryos whereas it is degraded in control embryos following fertilization [49]. 4E-BP mRNA is present as a maternal mRNA (Table 1). As shown in Figure 3, analysis of the mRNA distribution on sucrose gradient showed that 4E-BP mRNA is mostly associated with light fractions (3–5), showing no significant translation. The polysome profile of 4E-BP mRNAs in presence of mTOR inhibitor PP242 showed no increase of the mRNA level into polysomal fractions. This observation ruled out a possible contribution of newly translated protein to the 4E-BP pool, in addition to the already demonstrated PP242 inhibition of 4E-BP degradation rates [41,50].

**Figure 4:**
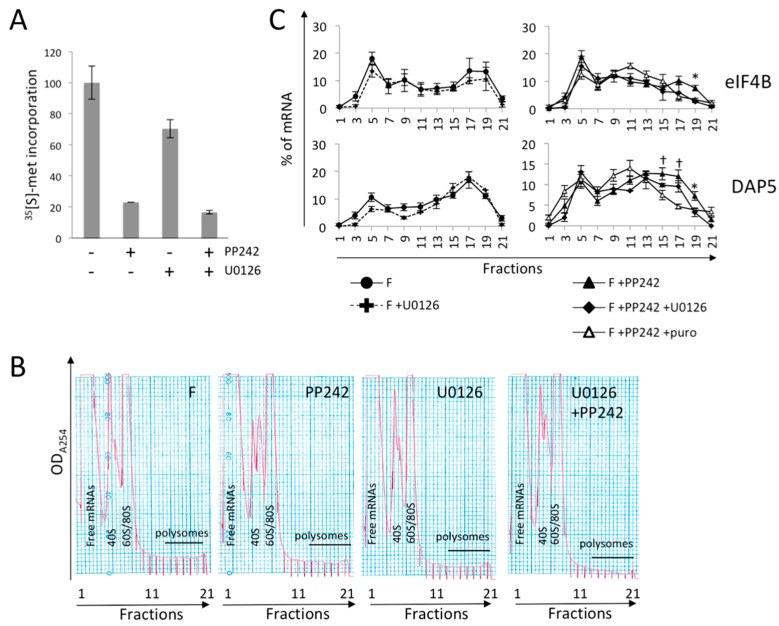
(**A**) mTOR inhibition and dual mTOR/MAPK inhibition impacts protein synthesis activity. Protein synthesis activity is measured by incorporation of [^35^S]-methionine in TCA-precipitated proteins, normalized to the values in fertilized control embryos (representative of two independent experiments). (**B**) Polysome profile of fertilized embryos (F) with or without PP242 and U0126 inhibitors. (**C**) Redistribution of mRNAs for eIF4B (**top**) and DAP5 (**bottom**) on polysome gradients, monitored as in Figure 1, in presence of U0126 (**left**) or in combination with PP242 (**right**). Values are shown as a mean of three biological replicates, error bars represent SEM (F+PP242 vs. F+PP242+U0126: * *p*-value < 0.05; F+PP242 +U0126 vs. F+PP242+puro: † *p*-value < 0.05).

We asked whether the remaining fertilization-induced polysome recruitment of eIF4B and DAP5 mRNAs could be dependent upon PP242-activation of the MAPK pathway. To test this hypothesis, PP242-treated fertilized embryos were incubated with the MAPK activation inhibitor U0126 [51]. Eggs were first treated with PP242 inhibitor 10 min before fertilization, U0126 was added 5 min after fertilization to prevent a possible reactivation of the MAPK pathway in response to mTOR inhibition, and both inhibitors were kept in contact with the embryos throughout the experiment. Protein synthesis was monitored one hour post-fertilization by [^35^S]-methionine incorporation. The mTOR inhibitor PP242 inhibited strong global translation activity, while the MAPK pathway inhibitor U0126 inhibited it moderately. Dual treatment of embryos weakly increased the inhibitory effect of PP242 on protein synthesis (Figure 4A). The distribution on polysome gradients of eIF4B and DAP5 mRNAs, exhibiting a residual translation in presence of PP242, was analyzed in three independent experiments. In embryos exposed to U0126 only, no significant variation in polysomal recruitment after fertilization was observed (Figure 4C left). When both PP242 and U0126 were present, eIF4B mRNA was no longer recruited to polysomes (Figure 4C right), suggesting that a MAPK activity relay is involved in its translation. In contrast, DAP5 mRNA recruitment was independent of mTOR activity and was still partially recruited when embryos were treated with both inhibitors, suggesting a MAPK relay and an additional regulatory mechanism, yet undetermined (Figure 4B right).

## 3. Discussion

We show in this report that the mRNAs coding canonical initiation factors eIF4E1, eIF4A, and eIF4G, as well as PABP and translation inhibitor 4E-BP, were not recruited into polysomes after fertilization. Conversely, mRNAs for eIF4B, which potentiates eIF4A activity, was recruited post-fertilization, suggesting that the mRNA unwinding activity could be activated after fertilization. Furthermore, mRNAs for non-canonical initiation factors such as eIF4E2, eIF4E3, DAP5, and hnRNP Q were recruited following fertilization. Unfortunately, no cross-reacting antibodies were available against the sea urchin proteins, therefore it was not possible to study the presence or accumulation of the proteins for which we have shown a translational regulation. The stored maternal initiation factors drive protein synthesis initiation for the first cell cycles; our results suggest that non-canonical initiation factors that are translated after fertilization may have a specific role later in development.

eIF4E2 and eIF4E3 are implicated in selective translation [18,19,20,21] rather than in global translation, which is driven by eIF4E1. During embryonic development in mice, *Drosophila*, and *Caenorhabditis elegans*, eIF4E2 acts as a selective translational repressor, replacing eIF4E1 [18,52,53]. In metazoan early development in general, and in sea urchin early development in particular, a redox gradient is responsible of the oral–aboral axis specification of the embryo [54,55]. A spatial rearrangement of mitochondria in the embryo confers an asymmetric distribution of oxygen availability and renders some embryonic regions hypoxic. eIF4E2 is able to drive protein synthesis in hypoxic conditions [56,57], when it associates to HIF2α and RBM4 to select the appropriate mRNAs to translate [19]. Although hypoxia induces 4E-BP reappearance in sea urchins [58], eIF4E2 may still mediate translation because it does not efficiently bind 4E-BP [15,48,59]. Furthermore, an RBM4 homolog mRNA is recruited into polysomes to a high extent after fertilization [45]. In our experiments, we observed the increased recruitment of eIF4E2 mRNA, suggesting a role of selective translation in sea urchin early development. Therefore, taken together, we suggest that newly translated eIF4E2 could be part of a regionalized non-canonical initiation complex and could drive spatial specific mRNA translation in hypoxic embryonic cells.

The initiation factor eIF4E3 binds to the cap in an atypical manner and binds less efficiently to eIF4G than eIF4E1 [15,21]. These features are responsible for the competition between eIF4E3 and eIF4E1 for eIF4E1 target mRNAs [21]. MNKs, the eIF4E1 kinases [60] regulated by the MAPK pathway, have a master role in the use of eIF4E1 or eIF4E3 to regulate mRNA recruitment in diffuse large B-cell lymphoma [20]. Inhibition of MNKs activity leads to an increase of eIF4E3 mRNA translation and to the preferential use of eIF4E3 as a cap-binding protein in these cells. In early sea urchin embryos, the MAPK pathway is physiologically inactivated at fertilization and peaks transiently at M-phase [51]. eIF4E3 has not previously been identified as a developmental eIF4E. Whether physiological MAPK inactivation is directly involved in the recruitment of eIF4E3 mRNA observed post fertilization and whether newly synthesized eIF4E3 is engaged into initiation complex formation in sea urchin development remains to be investigated.

A recent report showed that DAP5, through its interaction with eIF3d, also being a cap-binding protein [61], was able to drive the alternative cap-dependent translation of 20% of mammalian cell mRNAs [26]. Moreover, DAP5 was shown to promote IRES-mediated translation during mitosis [25,62]. Since the translation of some mRNAs, including DAP5, is not impacted by the mTOR inhibitor PP242, we suggest that DAP5 could participate in alternative cap-dependent and/or IRES-dependent translations in sea urchins. IRES-containing mRNAs have yet to be identified in sea urchins. In mammalian cells, hnRNP Q regulates the translation of several cellular IRES-containing mRNAs [30]; however, in sea urchins, its own translation is sensitive to the mTOR pathway. It would be interesting to investigate the hnRNP Q protein level in sea urchin eggs and embryos.

Overall, our results demonstrate that the polysomal recruitment of initiation factor mRNAs differs depending on their sensitivity to the mTOR pathway, ranging from completely dependent to completely independent. Our study also demonstrates the conservation of the mTOR and MAPK pathway relays in sea urchins [13,14] for the translation of specific mRNAs, namely eIF4B and DAP5 mRNAs.

In summary, the non-canonical initiation factors we identified as translationally regulated after sea urchin fertilization are all global protein synthesis repressors, and enhancers of selective translation. We suggest that these non-canonical eIFs could establish non-canonical translation initiation complexes enabling their translation selectivity. An interesting perspective would be to investigate, in parallel, the dynamics of the different initiation complexes and the maternal mRNA recruitment during the first cell divisions of early development up to the maternal-to-zygotic transition. These results highlight that the translation initiation process might be more complex than previously thought during the development of the sea urchin embryo.

## 4. Materials and Methods

### 4.1. Handling and Treatment of Eggs and Embryos

*Paracentrotus lividus* sea urchins were collected in the bay of Crozon (Brittany, France) and maintained in the CRBM facility of the Station Biologique de Roscoff. Gametes were obtained after intracoelomic injection of 1 mL acetylcholine 0.1 M. Unfertilized eggs were dejellied and rinsed before resuspension at 5% dilution in filtered sea water (FSW). Diluted sperm was added to the unfertilized eggs. Experiments were only performed on batches of embryos exhibiting >90% of fertilization rate. Embryos were collected for polysome analyses at 60 min post-fertilization. Inhibitors were added to the eggs or embryos at the indicated time points: PP242 [10 µM] at 10 min before fertilization; U0126 [60 µM], puromycin [0.6 mM], and emetine [0.1 mM] at 5 min, 40 min, and 55 min post-fertilization respectively.

### 4.2. Polysome Gradients and RT-PCR Analysis

Polysome gradients and their analysis were performed as described in [47]. Briefly, 250 µL of pelleted cells were lysed in a Dounce homogenizer with 1 mL polysome lysis buffer (10 mM Tris pH 7.4; 250 mM KCl; 10 mM MgCl_2_; 25 mM EGTA; 0.4% Igepal; 5% sucrose; 1 mM DTT; 10 µg/mL aprotinin; 2 µg/mL leupeptin; 100 µg/mL emetine; and 40 U RNase inhibitor). Lysates were clarified for 10 min at 13,000 rpm in a tabletop centrifuge. Supernatants were fractionated on a linear 15–40% sucrose gradient (10 mM Tris pH 7.4; 250 mM KCl; 10 mM MgCl_2_; 25 mM EGTA; and 1 mM DTT) for 2.5 h at 38,000 rpm in a SW41Ti rotor at 4 °C. Gradients were fractionated into 21 equal fractions. RNAs were extracted from each fraction using acid phenol–chloroform (*v*/*v*), precipitated with 1 volume isopropanol, washed with 70% ethanol, and resuspended in RNAse-free water. RNA quality was checked on a 2% agarose/TBE gel electrophoresis. Specific mRNA distribution along the polysome gradient was analyzed by semi-quantitative RT-PCR using an equal volume of RNA from each fraction as described [47]. Reverse transcription (RT) was performed using a constant volume of RNAs from each fraction with reverse transcriptase SuperScript II (Invitrogen, Courtaboeuf, France), following the manufacturer’s instructions. The resulting cDNA was diluted in RNase-free water (1 vol. RT/300 vol. H_2_O) for the PCR reaction, so that the amplification with each primer pair (see list in Table 1) was in the linear range for 30 cycles of amplification. Primers’ efficiency was determined using total RNA purified from eggs (except for eIF4E1 done on blastulae RNA). PCR were carried out with the GoTaq Flexi kit (Promega, Charbonnières-les-Bains, France) and [5 µM] primers, using the following thermal profile: 95 °C for 2min; followed by 30 cycles of 3 steps: 95 °C for 30 s, 60 °C for 30 s, 72 °C for 1 min; and finally, 72 °C for 5min. For each mRNA tested, a RT-PCR performed without reverse transcriptase was done in parallel to control for non-specific amplification. PCR products were analyzed on 2% agarose/TBE gels electrophoresis, scanned on a Typhoon Trio (GE Healthcare Life Sciences, Velizy-Villacoublay, France), and quantified with ImageJ software (NIH). Statistical analyses were done using a two-tailed Student’s *t* test.

### 4.3. In Vivo Protein Synthesis Analysis

Embryos (5% suspension in seawater) were taken one hour after fertilization and incubated for 15 min in 10 μCi/mL [^35^S]-l-methionine. [^35^S]-l-methionine incorporation into proteins was measured on duplicate aliquots after 10% TCA precipitation.

## Figures and Tables

**Table 1:** Translation initiation factors in the sea urchin maternal transcriptome (retrieved from [45]).

Name	Transcript #	FPKM *	Primer Sequence	Product Length (bp)	Efficiency (*R*^2^)
eIF4A	comp73316_c0_seq1	59.53	TGGTCAAGAAGGAAGAAC CGTCTCATACAAGTCACA	103	0.990
eIF4B	comp78411_c2_seq1	94.72	GGAGGAGCAAAGCCTGTAGA ACGCGTTCTGCTTTCTCTTC	200	0.992
eIF4E1	comp52193_c0_seq1	<1	GGTGGAAGGTGGCTCATAGG TCTTTCCTCCAGTCCCCTGT	191	0.996
eIF4E2	comp72071_c0_seq2	30	TATGGTCGGAGAGGAGAT ATTATTATCGCTGGCTGTG	128	0.989
eIF4E3	comp75131_c0_seq1	20.3	GTAAAGCCCCTATGGGAAGA TTGGTGCCCCTAATGCTTAC	185	0.996
eIF4G	comp69782_c0_seq2	2.6	CCATGTTGAGTGAGGATGCG ACCTTCTCCTGGGATCCTCT	225	0.979
DAP5	comp79103_c1_seq1	87.1	AGACGAGCAGGACCAGAGAG GTCGGCCTACAGTGGTGATT	205	0.994
PABP	comp73981_c0_seq1	6.5	GCACCTCAAGTTCGAGTTGG TGGTCTGGAAGTTAGGCTGG	201	0.992
hnRNP Q	comp75304_c0_seq1	1439.6	GAGGAGATGAACGGCAGAGA GTAGCCTCCAAAGTCCCTGT	230	0.999
4E-BP	comp78493_c0_seq1	13.2	CCCATGATTACAGCACTAC GGAAGTTACGGTCATAGATG	83	0.996

* FPKM: Fragments Per Kilobase of transcript per Million mapped reads.

**Table 2:** Features of canonical and non-canonical eIFs involved in mRNA recruitment.

Function	Protein	Interactions						Role and References
		Cap		eIF4G		4E-BP		
Cap-binding proteins	eIF4E1	+++		+++		+++		Canonical eIF [3,15,48]
	eIF4E2	+		/		+		Selective translation [15,18,19,20,21,48]
	eIF4E3	+		+		/		
Scaffolding protein		**eIF4E1**	**PABP**		**eIF3**		**eIF4A**	
	eIF4G	+++	+++		+		+	Canonical eIF [3]
	DAP5	/	/		+		+	Selective translation [24,25,26]
Poly(A)-binding protein		**poly(A)**			**eIF4G**			
	PABP	+++			+++			Canonical eIF [3]
	hnRNP Q	+			+++			Selective translation [28,29,30]
